# Yolo-pest: an optimized YoloV8x for detection of small insect pests using smart traps

**DOI:** 10.1038/s41598-025-97825-3

**Published:** 2025-04-23

**Authors:** Ayesha Hakim, Amit Kumar Srivastava, Ali Hamza, Muhammad Owais, Muhammad Habib-ur-Rahman, Salman Qadri, Mirza Abdul Qayyum, Fawad Zafar Ahmad Khan, Muhammad Tariq Mahmood, Thomas Gaiser

**Affiliations:** 1 Institute of Computing, MNS - University of Agriculture, Multan, 60000 Pakistan; 2 Crop Science, Institute of Crop Science and Resource Conservation (INRES), University of Bonn, 53115 Bonn, Germany; 3 Leibniz Centre for Agricultural Landscape Research (ZALF), Eberswalder Str. 84, 15374 Müncheberg, Germany; 4 Tropical Plant Production and Agricultural Systems Modelling (TROPAGS), University of Göttingen, 37077 Göttingen, Germany; 5 North Florida Research and Education Center, University of Florida, Gainesville, USA; 6 Institute of Plant Protection, MNS- University of Agriculture, Multan, 60000 Pakistan; 7 Department of Outreach and Continuing Education, MNS-University of Agriculture, Multan, 60000 Pakistan; 8 Department of Entomology, University of Georgia, Griffin, GA USA; 9 Department of Zoology, Cholistan University of Veterinary and Animal Sciences, Bahawalpur, 63100 Pakistan; 10 School of Electrical Engineering and Computer Science (SEECS), National University of Sciences and Technology (NUST), Islamabad, Pakistan

**Keywords:** Maize crop, Mango orchard, Pesticide use, Real-time, Object detection, Environmental protection andsustainability, Plant sciences, Mathematics and computing, Computer science

## Abstract

Fruit flies and fall-armyworm are one of the major insect pest that adversely affect fruit and crops, whereas fall-armyworm is also a highly destructive pest in maize crop but also damage other economically important field crops and vegetables. Adults of both pests can fly, making it hard to monitor them in the field. This study focuses on fine-tuning the YoloV8x model for automated monitoring and identifying insect pests, like fruit flies and fall-armyworms, in open fields and closed environments using IoT-based Smart Traps. The conventional techniques for monitoring of these insect pests involve pheromone attractants and sticky traps that require regular farm visits. We developed an IoT-based device, called Smart Trap, that attracts insect pests with pheromones and captures real-time images using cameras and IoT sensors. Its main objective is automated pest monitoring in fields or indoor grain storage houses. Images captured by smart traps are transmitted to the server, where Yolo-pest, a fine-tuned YoloV8x model with customized hyperparameters performs in real time for object detection. The performance of the smart trap was evaluated in a mango orchard (Fruit Flies) and maize field (fall Armyworm) in an arid climate, achieving a 94% average detection accuracy. The validation process on grayscale and coloured images further confirmed the model’s consistent accuracy in identifying insect pests in maze crop and mango orchards. The mobile application also enhances the practical utility as having a user-friendly interface for real time identification of insect pest. Farmers can easily acces the information and data remotely that empowering them for efficient pest maangment.

## Introduction

Agricultural productivity and quality significantly decrease due to fruit flies, causing 40–80% fruit loss depending on variety and season^1^. According to the UN Food and Agriculture Organization (FAO), the world needs to produce 70% more food by 2050 to meet the growing population’s demands under changing climatic scenarios^2–6^. There is a dire need to increase farm yields in the scenario of shrinking agricultural lands and resources^7–13^, and adaptation technologies are required for sustainable crops production to tackle negative imapcts of climate change and to meet food security and nutritional quality demands^14–17^.

The Fall Armyworm (*Spodoptera frugiperda*) is a highly destructive pest that primarily attacks maize (corn) but can also effect other economically important field crops^18–20^. It is widely distributed and causes serious damage to about 80 different commercial crops, including maize, rice, sorghum, sugarcane, cabbage, beet, groundnut, soybean, alfalfa, onion, pasture grasses, millet, tomato, potato, and cotton^12,16,20,21^. Fruit flies are one of the most harmful insect pests that affect crops through direct damage to the fruits^1^. The fruit fly infestation is a major hindrance to exporting fresh fruits and vegetables to international markets. *Bactrocera zonata*, *Bactrocera dorsalis*, and *Bactrocera cucurbitae* are common fruit flies in Pakistan^19,22^. They attack mango, banana, tomatoes, apples, oranges, and vegetables such as capsicum and squash. Different methods are used for managing fruit flies and fall armyworms (FAW) such as a cover spray of pesticides, baiting, and trapping. Pesticide application has several non-target effects on the beneficial fauna, soil and fruit quality, ecosystem and human health^23,24^.

Physical traps are used to monitor and manage fruit flies and fall armyworms by using baiting materials or pheromones as lures. For fall armyworm monitoring, manual pheromone traps are used to capture pest samples in maize field, indicating their presence^1^. Different trapping techniques, such as mass trapping and liquid protein trapping, can be employed. Mass trapping targets male fruit flies^19,22^, while liquid protein trapping focuses on capturing female fruit flies needing protein for egg development. These trapping methods are essential for assessing pest populations and implementing suitable control measures.

Traditional methods for monitoring fruit flies, such as pheromone traps and sticky tapes, necessitate regular manual visits to collect and count insects. Moreover, manual monitoring is time-consuming, labor-intensive, and often ineffective in timely pest management^19,20^. An alternative approach involves using Internet of Things (IoT) sensors for automated, real-time monitoring of fruit flies with minimal human intervention^25^. IoT sensors enable automated data collection, allowing continuous monitoring and reducing the need for manual visits. This approach greatly enhances the monitoring system’s performance and efficiency. Integrating IoT sensors enables real-time data collection, offering valuable insights into insect population dynamics^26–28^. This data is utilized for predictive analysis, enabling timely decisions in pest management^29^.

Evidence indicates several attempts at automating the classification of flying insect pests. For instance, in a recent study^22^an E-trap was proposed, which can be attached to a traditional polyethylene (PET) bottle and transmit images to a central tower in the field. This E-trap sends a single daily image to the central tower and processes it on an embedded system, which affects its accuracy in detecting fruit flies. Another approach^30^ introduced the Sticky-pi, which monitors and identifies insects captured using sticky tapeds. This trap is semi-automated, as the sticky tapes need regular replacement. In a study^31^, a plugin consisting of an ESP-32 microcontroller, an image sensor, and a microSD card for data storage was presented. The study suggested using it with customized pheromone traps for automated monitoring. However, the study lacks quality parameters or mitigation of other field environment limitations like overheating, fog, and dust. This plug-in is also semi-automated since it doesn’t provide real-time information on insect populations in the field, which is crucial for effective control measures. In the literature, there have been several attempts using YOLOv3 for detecting various insect pests^26,31^. A remote insect trap monitoring system^25^ was presented using a deep learning framework and IoT, achieving a 94% accuracy on a dataset containing 100 images of crawling and flying insects. In another study^32^ utilized a convolutional neural network (ResNet18) for counting and identifying Mediterranean fruit flies (*Ceratitis capitata)* with an accuracy of 90.7%, compared to a deep neural network (SqueezeNet) providing an accuracy of approximately 84.47%. Furthermore, researchers^33^ classified images of adult tephritid fruit flies, and classified images of insects *C. capitata* and South American fruit fly (*Anastrepha fraterculus)*, using image processing and machine learning. Literature revealed that studies have been conducted with different insect, pest, data set and environments like Chen et al.^34^ developed the Yolov4 algorithm for detecting pests (*Mealybugs*, *Coccidae*, and *Diaspididae)* and claimed to achieve 100% accuracy on the test dataset. It is worth noting that this dataset contains only 600 images, whereas the test dataset only contains 35 images. Likewise, Wen et al.^35^ proposed the Pest-YOLO model which was trained and evaluated on the Pest24 dataset, which includes more than 20,000 images with over 190,000 instances labeled and categorized into 24 classes. The results show that Pest-YOLO achieved 69.59% for mAP and 77.71% for mRecall on Pest24. In another study for maize crop, Yang et al.^36^ proposedx Maize-yolo which is designed to detect maize pests. The model was trained and evaluated on the IP13 dataset which contains only 4533 images. Maize-yolo achieved an accuracy of 0.763 mAP. All these approaches have been evaluated with limited number of images of insects captured in controlled environments, and their accuracy has not been tested in the field.

On a commercial scale, there are a few traps available in the international market for the automated monitoring of insect pests. For example, SnapTrap in Australia^37^ counts captured fruit flies but that is expensive for farmers in developing countries. It counts pests without species and location identification, making it challenging to make decisions about pesticide use at specific locations. Another option available is RapidAim^38^, which claims to provide real-time information about fruit fly pest detection in orchards and farms. However, it does not perform hotspot identification and relies on detecting insects based on their characteristic movements rather than appearance. TrapView^39^ is also designed to detect larger insect pests such as moths. An electronic trap with optoelectronic sensors was developed by Potamitis eta al^40^ to monitor insect movement and wingbeats but does not analyze the captured recordings. None of these tools are versatile enough to be used in various field environments, including extreme temperatures, dust, fog, and have limitations such as low internet connectivity, and power backup. The proposed smart trap is an Artificial Intelligence (AI) and IoT-based device for real-time monitoring, species identification, and counting of flying insect pests (FIPs) that attack major fruit and vegetable crops. It can monitor and control FIPs such as fruit flies, mango hoppers, thrips, pink bollworms, moths, aphids, and locusts that attack various crops. Specifically, the current study focus on the fall armyworm and three fruit fly species: *B. zonata*, *B. dorsalis*, and *B. cucurbitae*. The smart trap is designed and tested under varying field conditions, including extreme temperatures, low connectivity, and power backup. A weather station with temperature and humidity sensor assembly measures local air temperature and humidity in the field. The time series data of temperature, humidity, and insect count helps to monitor the effect of changing weather on the population density of insect pests in the field. The smart trap aims to automatically monitor fruit flies and fall armyworm adults in open fields and provide real-time alerts through a mobile application. The objectives of this study include, (i) designing customized traps with embedded weather stations for fruit fly and fall armyworm population monitoring using cameras and IoT sensors, (ii) training deep learning models based on images of fall armyworm and fruit fly species, and (iii) developing a mobile application to generate alerts and notifications for farmers.

## Materials and methods

### Trap housing

**Fig. 1:**
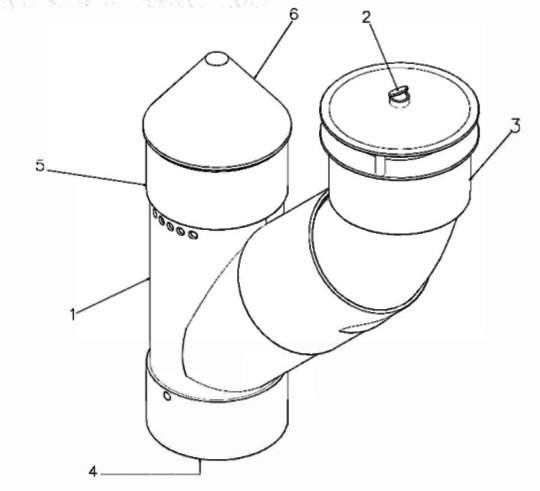
Isometric view of the smart trap.

The housing of the Smart Trap device is made up of a curved rigid Polyvinyl chloride (PVC) to keep it safe from weather and other environmental hazards. Figure 1 presents a comprehensive depiction of the essential components (1 to 6) central to the smart trap system. At the core of the design is the Main Chamber (1), serving as a foundational structure for capturing flying insect pests. Equipped with at least one intake, the Main Chamber acts as a hub for captured insects. The Pheromone Chamber (2), intricately connected to the Main Chamber, is configured strategically to contain, and release species-specific pheromones. These pheromones function as potent attractants, playing a pivotal role in enticing targeted insects into the trap. Situated above the Pheromone Chamber is the Funnel (3), well placed to guide insect traffic. The Funnel serves as the primary entry point for pests, effectively preventing certain undesired insects from entering the Pheromone Chamber.

In the same illustration, the Base Plate (4), securely attached to the bottom of the Main Chamber, functions as a designated collection area for captured insects. This design streamlines the retrieval and monitoring process. It can be automatically rotated to clean up the base plate. Highlighted in Fig. 1 is the Electronic Sub-System Chamber (5), mechanically connected to the Main Chamber. This chamber emerges as a pivotal component, housing advanced technology and devices crucial for collecting and processing vital data. Emphasized in the illustration is the Protective Cap (6), installed atop the Main Chamber. Serving as an indispensable element, the cap provides a protective barrier for the Electronic Sub-System Chamber (5), safeguarding it against external elements and potential tampering. Figure 1 collectively portrays the seamless integration of these components, illustrating the systematic design and functionality that defines the smart trap system.

**Fig. 2:**
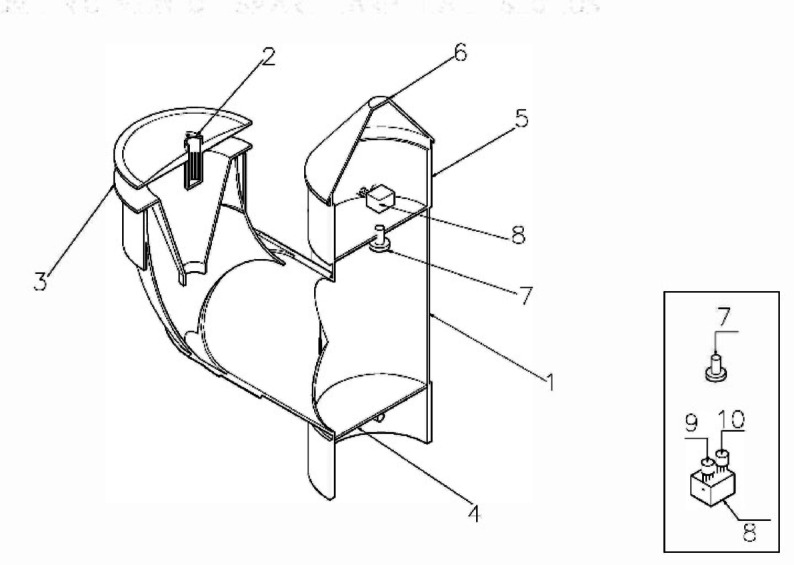
Cross-sectional view of smart trap system.

Figure 2 provides a cross-sectional view of the trap, revealing key components (7–10) crucial to the system’s functionality. Positioned above the Base Plate (4) within the Electronic Sub-System Chamber (5), reference numeral (7) designates the High-Resolution Camera. This camera, equipped with a wide-angle lens, captures real-time images and videos of trapped insects, offering valuable visual data for comprehensive monitoring. Adjacent to the camera, the Sensor Assembly (8) within the Electronic Sub-System Chamber (5) includes at least one Temperature Sensor (9) and one Humidity Sensor (10). These sensors play a critical role in collecting essential environmental data related to temperature and humidity, aiding in the understanding of insect behaviour and population dynamics. Figure 2 collectively unveils the sophisticated integration of these elements, highlighting the intricate design and advanced monitoring capabilities of the smart trap system.

### Trap functionality

A GSM-based device is used to provide internet connectivity to the trap for transmission of images to the server. To ensure an uninterrupted power supply to the smart trap, a solar photovoltaic (PV) system backed up by rechargeable lithium batteries is used.

An 18-watt controller with 2 USB ports was used as an interface between the Solar PV system and the battery, containing 12 lithium cells, along with a buck converter and a 3 S battery management system (BMS) to ensure longer battery life and power backup. A chemical lure was placed in the Pheromone Chamber to attract insects. To attract the selected species of fruit flies (B. zonata, B. dorsalis, and B. cucurbitae) towards the smart trap, soaked cotton wool with the natural chemical compound methyl eugenol, a recommended attractant for fruit flies, was employed to enhance capture efficiency. To attract fall armyworms, a commercially available FAW lure was utilized. Methyl eugenol can attract fruit flies from around a 20 m radius, while FAW lure can attract fall armyworms from a 35 m radius.

The trap is designed with a funnel-shaped entrance leading to the Main Chamber that allows insects to enter but makes it difficult for them to escape. Upon entering the trap through the funnel-shaped opening, pests are directed to the base plate, where an additional set of pheromones is placed to enhance retention of the captured specimens. This method ensures that once the pests are attracted into the trap by the initial lure at the entrance, the additional pheromones inside discourage them from attempting to leave, increasing the likelihood of their capture. This strategy can be particularly useful in maintaining the efficiency of the trap over extended periods, maximizing the capture rate of target pests. The trapped insects eventually die of starvation. However, some pests may survive for several days, the detection system is designed to minimize errors caused by repeated identification of the same pest. The aim of the detection system is to calculate the Economic Threshold Level (ETL) for pest management. The ETL is determined by analysing multiple images captured over a day, with the average pest count from the images being used to estimate pest population density. Moreover, the heat generated by the internal components of the trap increases the temperature inside the pest chamber, reducing the survival rate of fruit flies. Survival time decreases significantly at temperatures above 35 °C, especially when the humidity is below 70% and there is no food available. Under these conditions, fruit flies can only survive for approximately 2–3 h^41^.

**Fig. 3:**
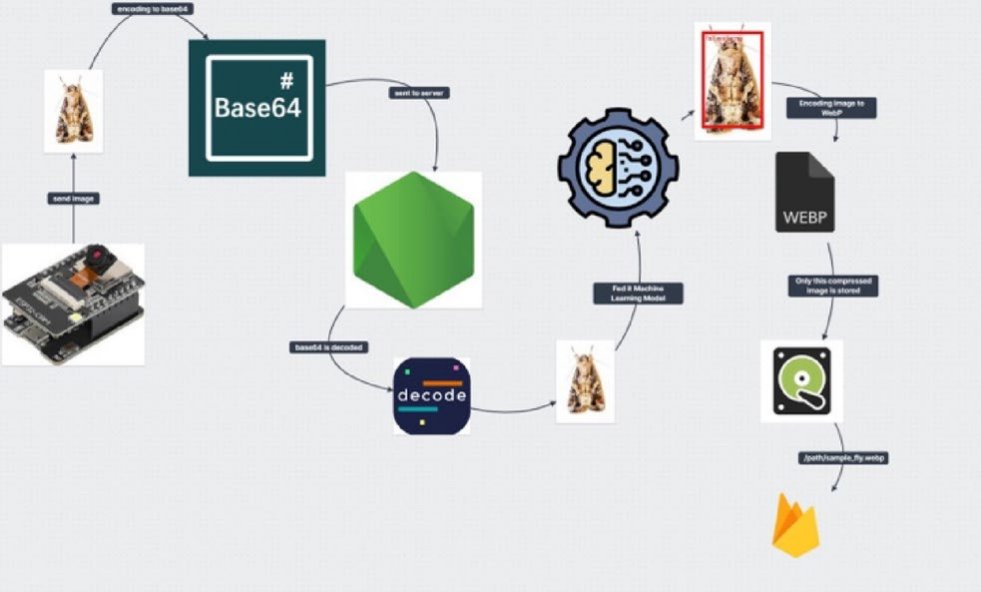
Image request life cycle from the smart trap (client) to the server.

As insects accumulate inside the chamber during infestation, the resulting pile complicates species detection for the machine learning model, reducing its accuracy and effectiveness in classification. To resolve this issue, a servo motor is attached to the base plate which is used to rotate and throw away all dead insects on the ground after regular time intervals. The data of user configuration, trap profiles, precise humidity, temperature, and date/time are stored and updated in the Firebase real-time database after regular intervals of time. Images captured by the camera are converted to base64 format at the client end that are then transmitted to the server. The specification of the server is Ampere A1 4 Cores (ARM64), 24 GB RAM, 2 Gbps Bandwidth, and 80 GB Storage. The server decodes base64 back into the jpeg format and feeds it as input to the deep learning model. If the model detects any targeted insect pest in the image, the server converts that image into webP compression. WebP is a modern image format developed by Google that provides both lossless and lossy compression for images on the web. It is designed to offer smaller file sizes without significant loss of image quality. The URL to this webP file is stored in Firebase firestore for history records. The complete image request life cycle is shown in Fig. 3. If the detected insect is one of the targeted pests, it will be notified to the farmer along with the details about insect species, location, date/time, temperature, and humidity values through a customized mobile application.

### Dataset

**Fig. 4:**
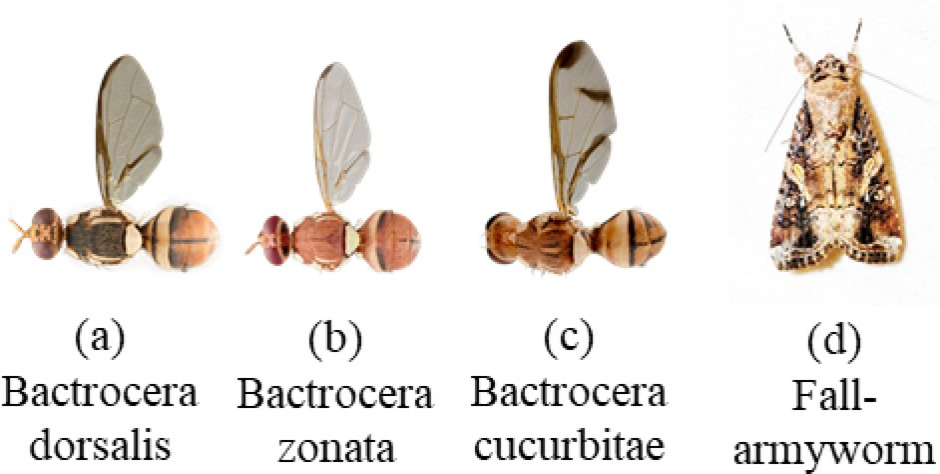
Selected flying insect pests in the dataset^42^.

FAW and fruit fly image datasets were collected from two sources: first by rearing insects in a controlled laboratory environment for image acquisition, and second by capturing images using a smart trap camera deployed in the field. A portion of this dataset is publicly available online^43^. *B. dorsalis* has yellow markings on the thorax and a dark T-shaped marking on the abdomen. *B. dorsalis* contains the complete coastal band and complete line on the abdomen (Fig. 4a), while *B. zonata* is radish brown in color with an apical spot on the end of the wings (Fig. 4b) It does not contain a costal band on the wings, *Bactrocera cucurbitae* is identified by the absence of yellow markings on the thorax, a dark T-shaped marking on the abdomen, and the presence of a complete costal band and line on the abdomen, it also exhibits distinct wing patterns characterized by a combination of dark markings and a complete costal band wings (Fig. 4c). Fall armyworm moths can be easily distinguished by their shape, size, and unique wings pattern (Fig. 4d).

**Table 1:** Augmentation techniques with specified values applied to the dataset.

Augmentation	Value
90° rotate	Clockwise, counter clockwise
Blur	Up to 2px
Brightness	Between − 25% and + 25%
Exposure	Between − 10% and + 10%
Flip	Horizontal, Vertical
Grayscale	5% of the total dataset
Hue	Between − 30° and + 30°
Noise	Up to 2% of pixels
Rotate	Between − 22° and + 22°
Saturation	Between − 20% and + 20%
Shear	±8° Horizontal, ± 16° Vertical

**Fig. 5:**
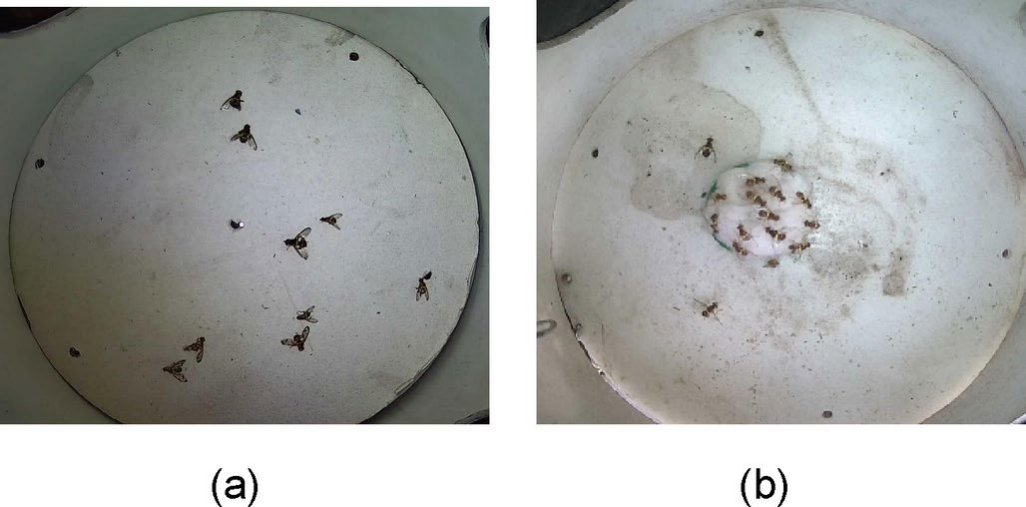
(**a**) Fruit fly image captured in the laboratory by rearing. (**b**) Fruit fly image captured in the field using a smart trap camera.

**Fig. 6:**
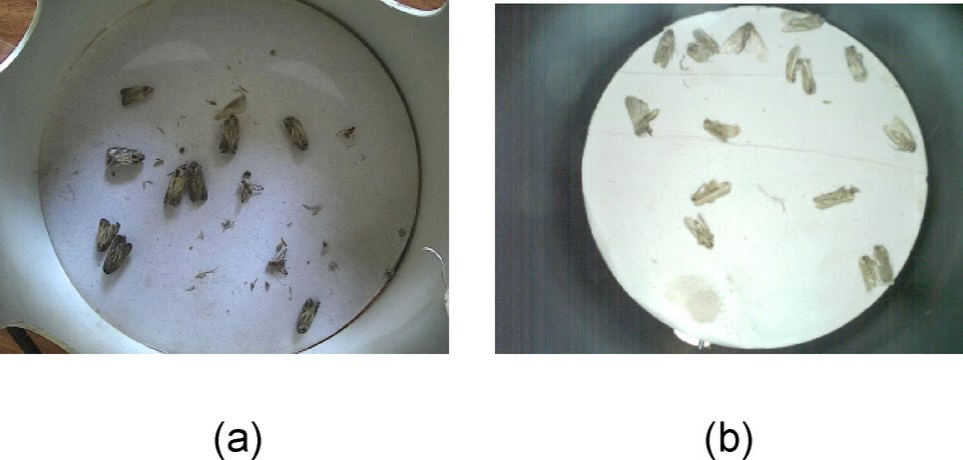
(**a**) Images captured in the laboratory by rearing fruit flies and fall-armyworm. (**b**) Image of fall-armyworm captured in the field using smart trap camera.

Figure 5a shows the images of dead fruit flies reared in the laboratory. Figure 5b shows the images of fruit flies taken using a customized IP camera (5-megapixels) that was connected to the smart trap installed in the mango orchard. Figure 6a shows the images captured in the laboratory by rearing fall-armyworm. Figure 6b shows the image of a fall armyworm captured in the field using a smart trap camera.

**Fig. 7:**
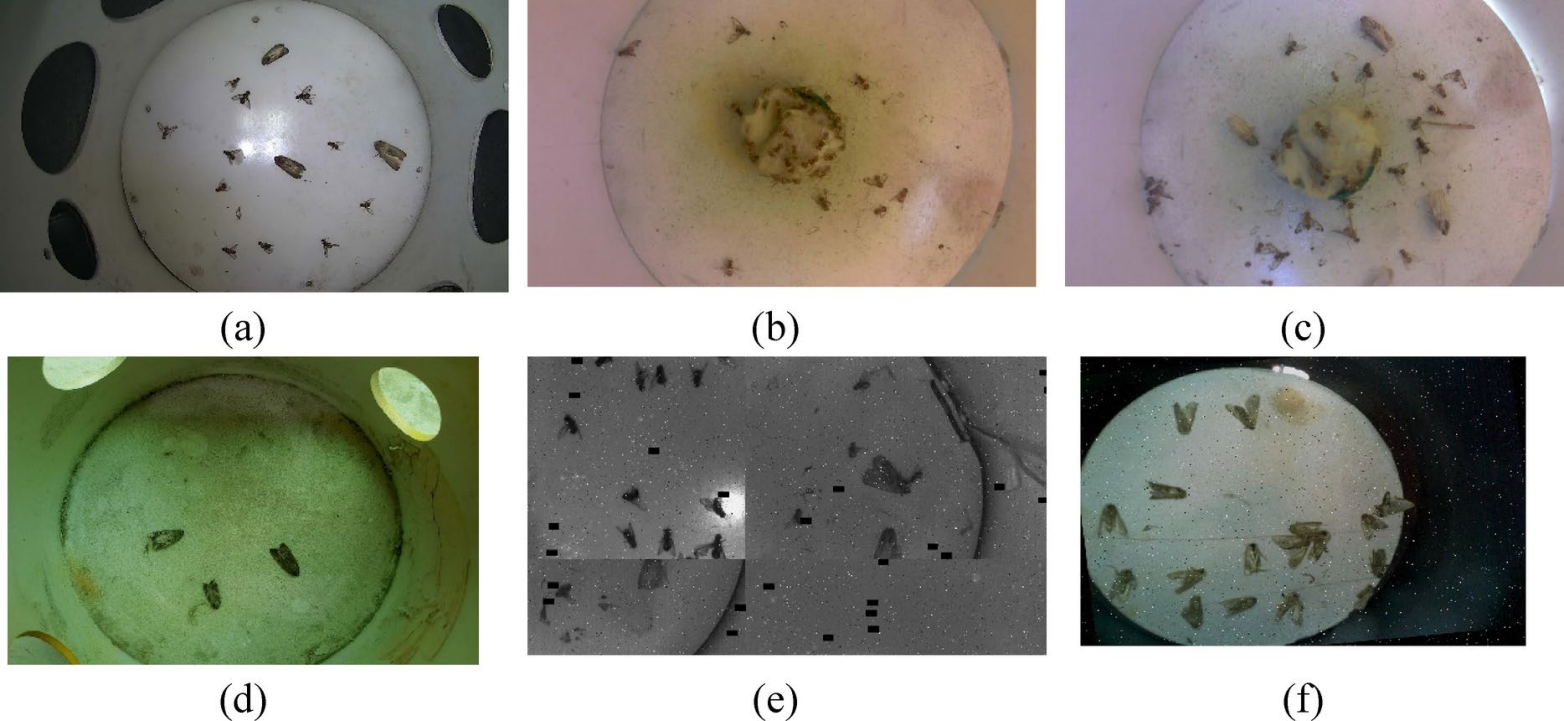
Sample images from FourPest dataset, (**a**) Image of fruitflies and fallarmyworm at night time. (**b**) Image of fruitflies in the field in day time. (**c**) Image of fruitflies and fallarmyworm with added noise and dribs, (**d**) Image of fallarmyworm in the lab, (**e**) Mosaic of four images of fruitflies and fallarmyworm with grayscale and cutout applied. (**f**) Image of fallarmyworm in the trap.

**Fig. 8:**
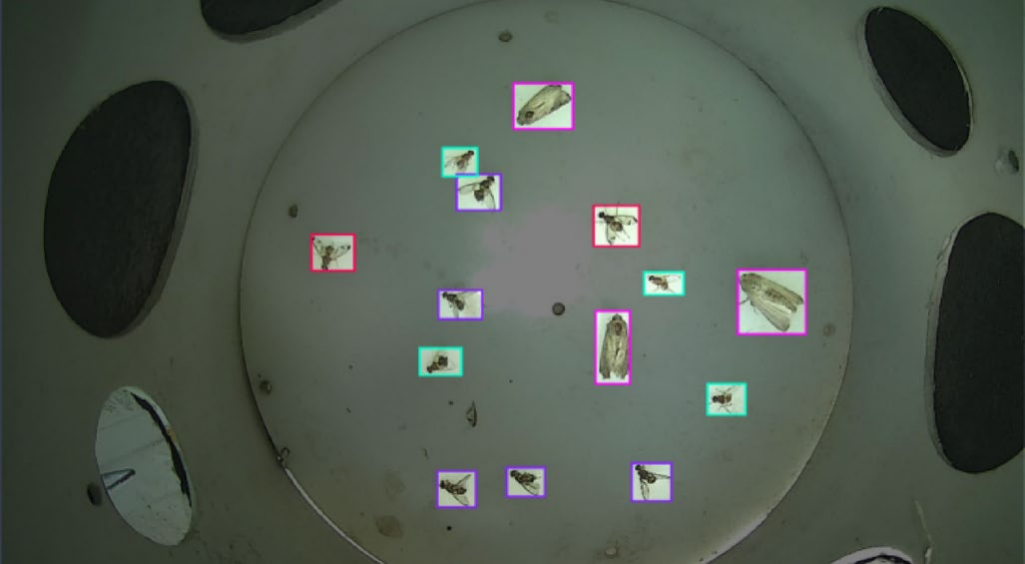
Smart trap image annotated as ‘dorsalis’,‘zonata’,‘cucurbitae’, and ‘fall-armyworm’ using roboflow bounding box.

Smart traps were tested under diverse environmental conditions such as day and night-time, images with noisy and polluted base plates, images captured in the lab, mosaic of images with grayscale and cut-out backgrounds, and images captured in the field in uncontrolled environmental conditions. Figure 7 shows a few instances of diverse environmental conditions where the smart traps were deployed. The dataset was labeled using Roboflow, which is a graphical image annotation tool, by drawing bounding boxes around each insect and labeled them as ‘**dorsalis’**, ‘**zonata’**, ‘**cucurbitae’** or ‘**fall-armyworm’** in yolov8 labeling format as illustrated in Fig. 8. In yolo8 labeling format, a text file with the same name was created for each image file in the same directory that contains the annotations for the corresponding image file. The annotation includes the class name, coordinates, height, and width of the annotated object.

**Fig. 9:**
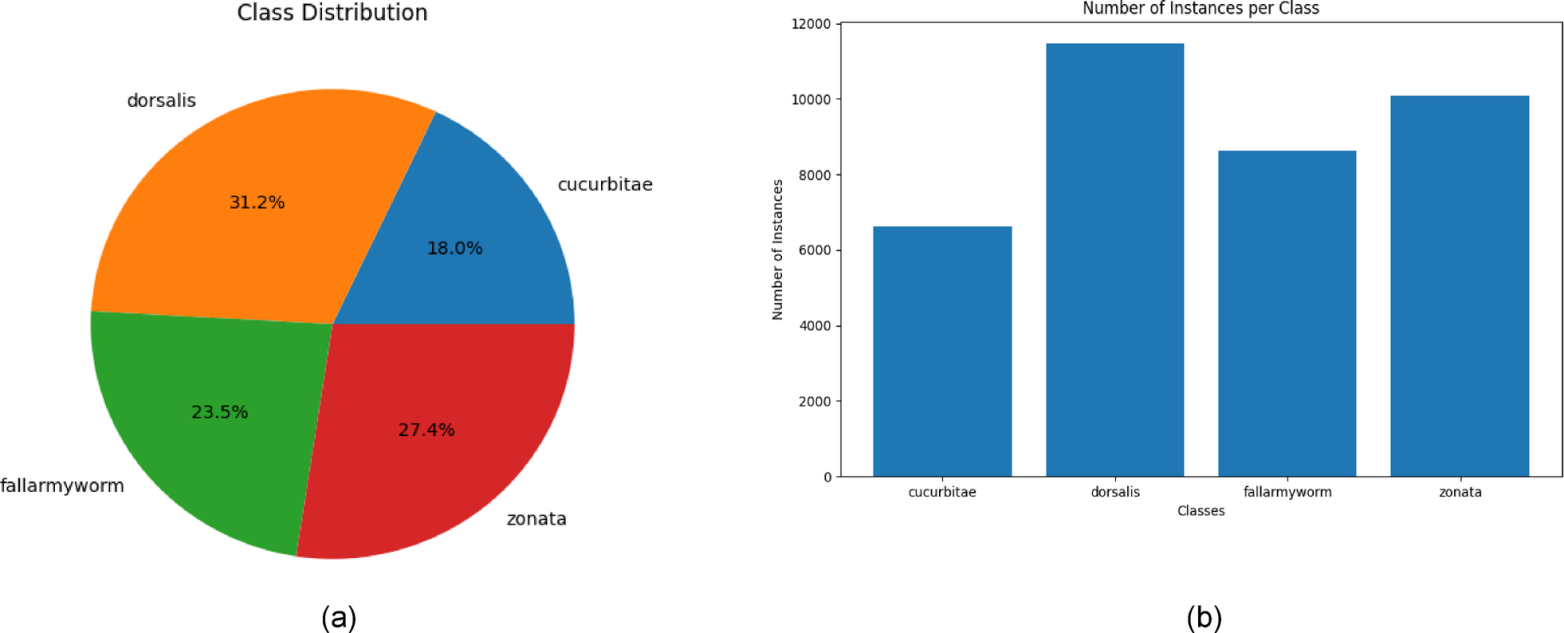
(**a**) Showing the distribution of class instances in dataset. (**b**) Number of instances in each class of dataset.

After data annotation, the dataset was divided into separate training, validation, and testing subsets, each of which contained 70%, 20%, and 10% of the total images. Image augmentation methods were applied to expand the training set and improve the model’s generalization capabilities. The detailed list of augmentation techniques applied to the dataset is shown in Table 1. The reliability of the object detection dataset depends not only on the diversity of distinct images but also on the number of instances within the dataset. The dataset was named “FourPest.” collected from both rearing and field sources contains 36,822 instances (cucurbitae: 6619; dorsalis: 11472; zonata: 10095; fall-armyworm: 8636), Fig. 9a & b representing the distribution of four classes on number of instances. The number of instances is varied due to the lack of availability of cucurbitae samples. The variance in the number of instances is attributed to the scarcity of cucurbitae samples. Recognizing the importance of addressing the class imbalance, label smoothing was employed as a strategic solution^44^. This technique not only mitigates the impact of the limited cucurbitae samples but also serves as a mechanism to prevent the model from overfitting to the existing labels.

By introducing a degree of uncertainty in the training labels, label smoothing encourages the model to learn from the underlying features, fostering a more robust and generalized performance.

The label smoothing formula is as follows:

1 $$\:Smooth\:label=\:true\:label\:\:\left(1\:-\:\epsilon\:\:\right)+\frac{\epsilon\:}{n}whereas,\:\epsilon\:=0.1,\:\:n=4$$

The degree of smoothness was set to 0.1 which is represented by epsilon ($\:\epsilon\:)$ in Eq. (1), the *true label* represents the predicted class whereas $\:n$ represents the total number of classes in the dataset. This formula blends the true class probability with a small amount of probability $\:\frac{ϵ}{n}$ distributed among other classes. This ensures that the model doesn’t become too certain about its predictions and encourages it to generalize better.

**Table 2:** Parameters used for training dataset ‘FourPest’ comprising of 3 fruit fly species and FAW.

Parameters	Value
epochs	200
patience	40
batch	36
imgsz	800
cache	ram
pretrained	true
optimizer	auto
close_mosaic	35
iou	0.96
max_det	550
label_smoothing	0.1
kobj	1.5

## Deep learning architecture

The cutting-edge YOLOv8x deep learning architecture was employed, customized with parameters detailed in Table 2, for the trap system implementation. This architecture boasts 365 layers and a substantial parameter count of 68, 229, and 648, facilitating precise object detection. The system is adept at classifying both fruit flies and Fall Armyworms by analysing various visual characteristics, such as colour, size, shape, and patterns found on their wings, abdomen, and thorax.

**Fig. 10:**
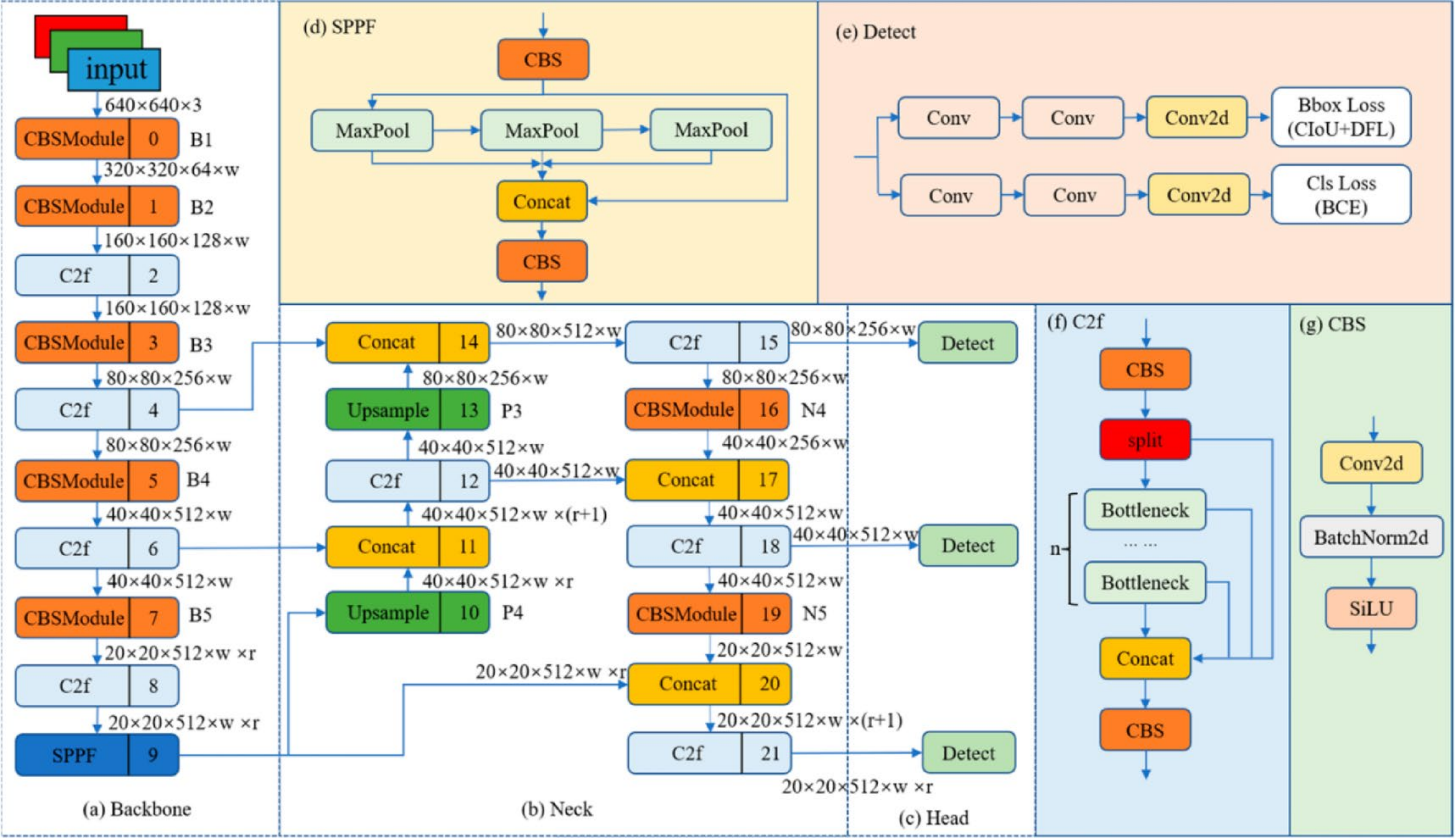
The network structure of Yolo-pest (Image taken from Wang et al.^45^)

The detailed diagram of yolov8x architecture is presented in Fig. 10. The backbone network of YOLOv8x uses a modified version of CSPDarknet53^45^ to extract features from the input image. Then, these features are down-sampled five times to produce various scales of features, designated B1 through B5. A C2f module (see Fig. 10f) improves feature extraction while keeping the model lightweight, and is also a part of the backbone network. Extracted features are processed by convolution, batch normalization, and activation using the CBS module. The feature maps are compressed into a fixed-size map using the spatial pyramid pooling fast (SPPF) module shown in Fig. 10d. A PAN-FPN structure, used by YOLOv8x in the “Neck” section shown in Fig. 10b, oversees combining and enhancing the features from various scales. Through feature fusion, this structure improves both semantic and positional information. The feature pyramid structure is a popular technique for detecting tiny objects by combining features at various scales^46^.

YOLOv8 uses a decoupled head structure in the “Head” section shown in Fig. 10c and d, to perform the small object detection task. This indicates that it divides the process of classifying objects and determining their bounding boxes into separate branches. YOLOv8 uses complete intersection over union (CIoU) and distribution focal loss (DFL) to predict the position of object-bounding boxes to increase prediction precision and training effectiveness. As an anchor-free detection model that more effectively specifies positive and negative samples, YOLOv8 is exceptional. Furthermore, it dynamically assigns samples using the Task-Aligned Assigner which further improves overall accuracy and robustness. The training process is configured to run for up to 200 epochs, but it will automatically halt if there is no improvement in performance metrics for 40 consecutive epochs. Given that the labeled instances are relatively small compared to the entire image, the default image size of YOLOv8x is adjusted to 800 pixels to enhance accuracy. Additionally, the batch size is set at 36.

**Fig. 11:**
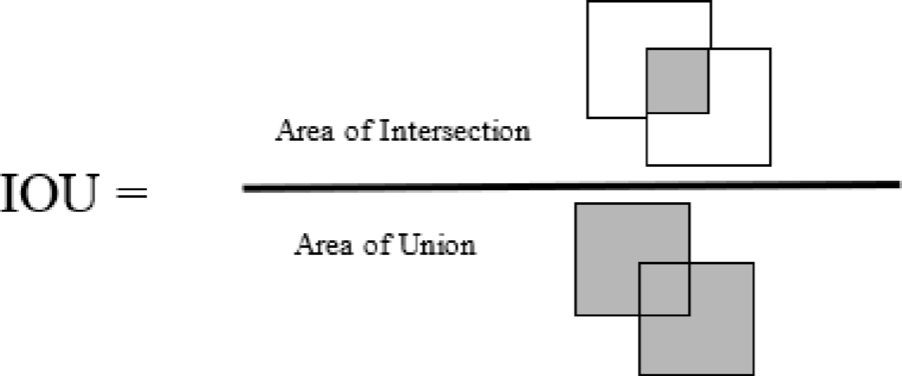
IOU used in non-maximum suppression (NMS).

To increase the training speed RAM cache is used. Yolov8x.pt model weights were used as a pre-trained model for transfer learning. The Yolo architecture introduced mosaic augmentation techniques which is a powerful method for extracting features from images This technique involves combining multiple images into a single mosaic image to create a new, augmented representation. While mosaic augmentation is beneficial for training, it may marginally impact the model’s performance on testing and validation datasets. To mitigate potential issues during the final stages of training, the *close_mosaic* argument is introduced with a value of 35. This parameter is employed to cease the application of mosaic augmentation in the last 35 iterations of training, aiming to ensure that the model’s performance is not compromised during the crucial testing and validation phases. Most pests are swarmer; they overlap each other, while objects overlap each other; this can make deep learning models difficult to recognize as their shape is distorted in the image. To overcome this problem, YOLOv8x uses a deep learning technique IOU threshold as shown in Fig. 11.

**Fig. 12:**
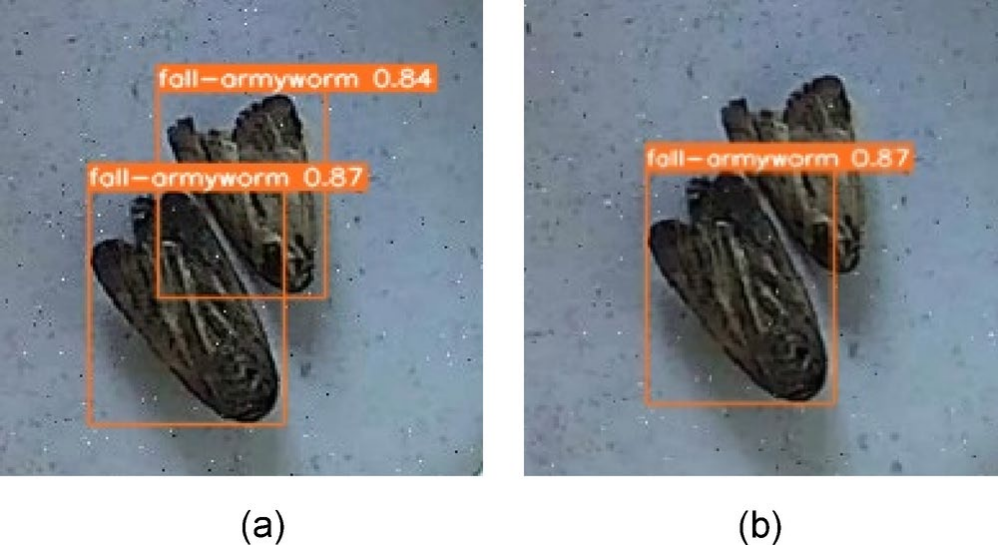
(**a**) iou-thres value 0.9. (**b**) iou-thres value 0.6 (Default Value).

The IOU threshold is one most common object detection techniques that is used to join the intersecting boundary boxes^47^. The default value of *iou-thres* is 0.6, which means bounding boxes of the same class that overlap each other by more than 60% are joined in Non-Maximum Suppression (NMS). The value of IoU threshold was increased to 0.9, and the maximum number of bounding boxes drawn per image *(max_det)* was elevated to 550. Figure 12a shows the predicted objects after NMS with the value of 0.9 threshold, which means in NMS, the algorithm will only join bounding boxes if they overlap more than 90%. Figure 12b shows the prediction of the same model with the default value of 0.6.

The fruit flies are nearly identical and even recognizing them from human eyes requires effort, slightly blurred images or human error in labels can prone the accuracy of CNN models. Label smoothing has proven to be a valuable strategy in this scenario, preventing excessive confidence in model inferences and promoting greater generalization.

The auto optimizer feature was utilized to automatically select the optimal optimizer and its associated parameters, including momentum, learning rate, and decay. In this specific scenario, the ‘auto’ optimizer feature of YOLOv8x identified Stochastic Gradient Descent (SGD) for the training process. The training process was conducted using the Stochastic Gradient Descent (SGD) optimization algorithm, with a learning rate set at 0.01 and a momentum of 0.9. The training involved distinct parameter groups: 97 weights with zero weight decay, 104 weights with a weight decay of 0.000338, and 103 biases with zero weight decay.

## Training and evaluation

The object detection model YOLOv8x was trained on Google Colab Pro + utilizing an Nvidia Tesla A100 GPU, which provided sufficient RAM and significantly enhanced the training speed of the model. Object detection is a computer vision technique that helps to identify and locate the objects in an image. This technique creates bounding boxes around the object of interest to locate the detected object among multiple objects. The model classified images into four classes namely ‘cucurbitae’, ‘dorsalis’, ‘zonata’, and ‘fall-armyworm’. Initially, Training was conducted for 200 epochs; however, the model achieved convergence after 100 epochs. Consequently, training was halted at 140 epochs, employing a patience of 40 epochs. After 140 epochs, the best.pt file was obtained, achieving a mean Average Precision (mAP) of 0.93, as presented in Table 3.

The evaluation metrics used to measure model accuracy were Precision, Recall, and mAP. Equation (2) defines the Precision metric where TP represents True Positive and FP represents False Positive. Equation (3) defines Recall metric (R) where FN represents the False Negative, Eqs. (4) and (5) define mAP which is used to calculate the accuracy of the model whereas, $\:p$ represents the precision, $\:r$ represents the recall and $\:n$ represents the total number of classes and $\:t$ represents a specific class from the dataset.

To measure the performance of the object detection model, precision, and recall were used for the calculation of the F1-score. Precision is the division of true positive results and the number of all positive results. The harmonic mean of precision and recall refers to the F1-score. The precision-recall curve is a trade-off between precision and recall at different values.

**Table 3:** The performance of the Yolo-pest model on the testing set with an overall 0.93 mAP.

Class	Box (*P*)	(*R*)	mAP50	mAP(50–95)
All	0.8415	0.963	0.9317	0.739
Cucurbitae	0.921	0.985	0.956	0.749
Dorsalis	0.827	0.948	0.916	0.7
Fall-armyworm	0.861	0.99	0.978	0.857
Zonata	0.757	0.93	0.877	0.653

2 $$\:P=\frac{TP}{TP+FP}$$

3 $$\:R=\frac{TP}{TP+FN}$$

4 $$\:AP=\:{\int\:}_{0}^{1}p\left(r\right)dr$$

5 $$\:mAP=\frac{1}{n}{\sum\:}_{t=1}^{n}{AP}_{t}$$

**Fig. 13:**
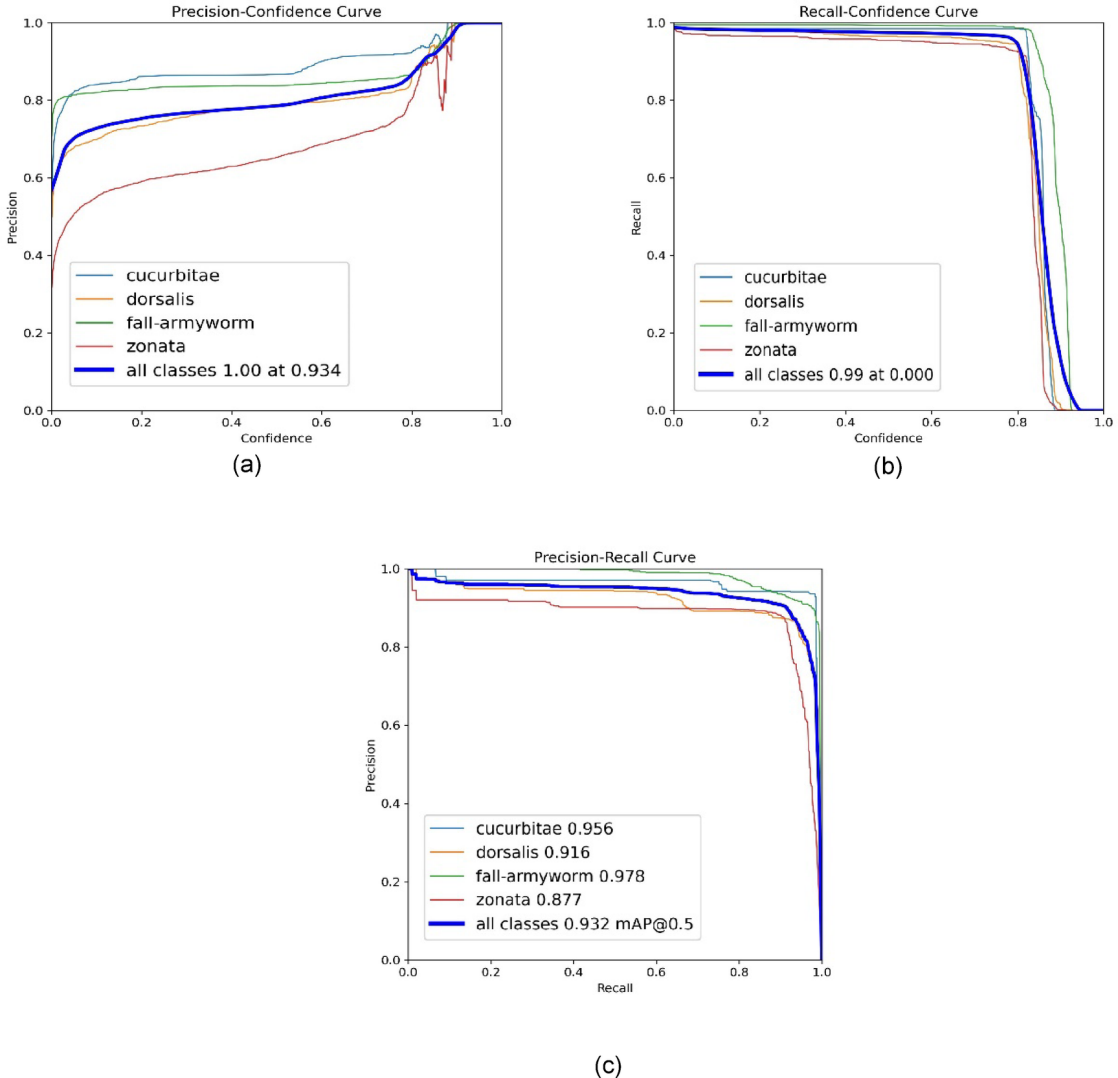
(**a**) Model Precision curve. (**b**) Model Recall curve. (**c**) Precision-Recall curve.

After getting results for training, the trained model was evaluated on the testing dataset and achieved an overall mAP of 0.974 as illustrated in Table 4. The table lists instances of all classes and respective Box (P) and Box (C). Box (P) represents the predicted boxes that represent the number of bounding boxes that the model has predicted for objects in the image. Box (C) represents the correctly predicted boxes that represent the subset of the predicted bounding boxes that have been correctly identified and localized, meaning they have a high overlap with the ground truth or annotated boxes. Figure 13a–c present graphs showing the precision curve, recall curve, and precision-recall curve of the model in the detection of each class individually and all classes combined.

**Fig. 14:**
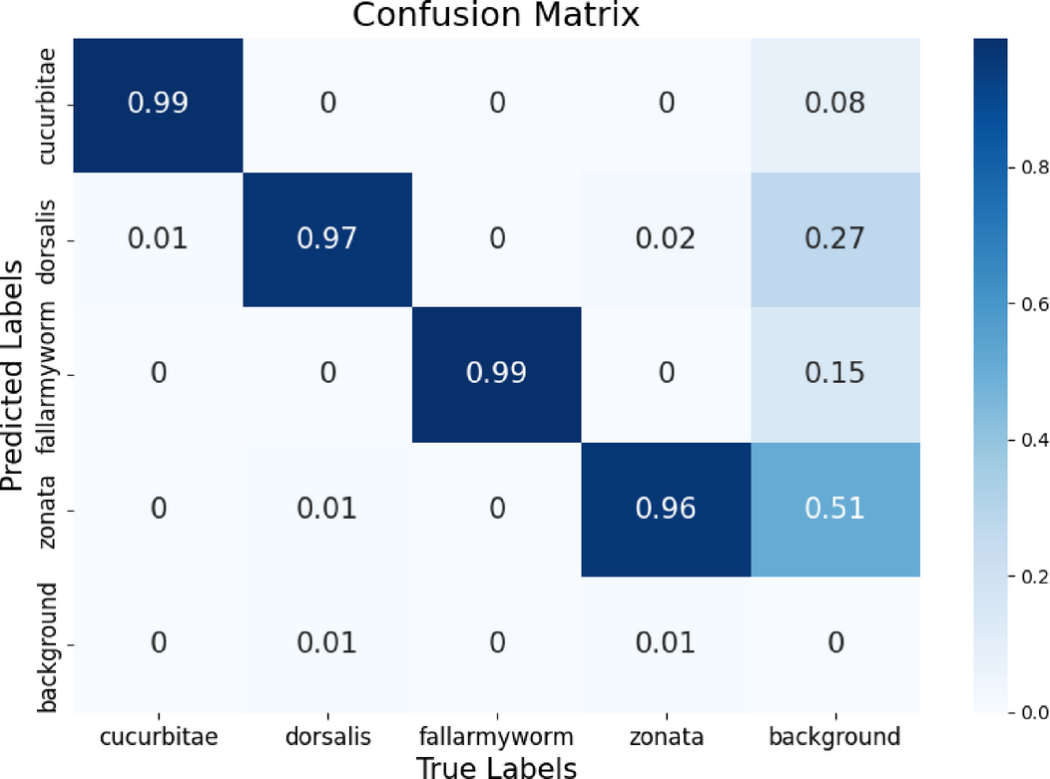
Normalized Confusion Matrix of Yolo-pest representing actual versus predicted values of Bactrocera dorsalis, Bactrocera zonata, Bactrocera cucurbitae, and Fall-Armyworm.

Figure 14 presents the normalized confusion matrix based on actual versus predicted values. The true-positive value of *Bactrocera dorsalis* is 97% and the false-positive value of dorsalis is 3%. In the case of *Bactrocera zonata*, the false-negative value is 4% and the true negative value is 96%. Due to the distinct wing patterns of *fall-armyworm* and *Bactrocera cucurbitae*,* the* true-positive value is 99% and the false-positive is only 1%.

**Table 4:** The performance of Yolo-pest on the testing set with an overall 0.974 mAP.

Class	Instances	Box (*P*)	Box (C)	mAP50	mAP(50–95)
All	1580	0.965	0.976	0.974	0.776
Cucurbitae	543	0.991	0.986	0.987	0.777
Dorsalis	922	0.951	0.972	0.972	0.757
Fall-armyworm	779	0.976	0.997	0.995	0.874
Zonata	858	0.942	0.949	0.941	0.693

**Fig. 15:**
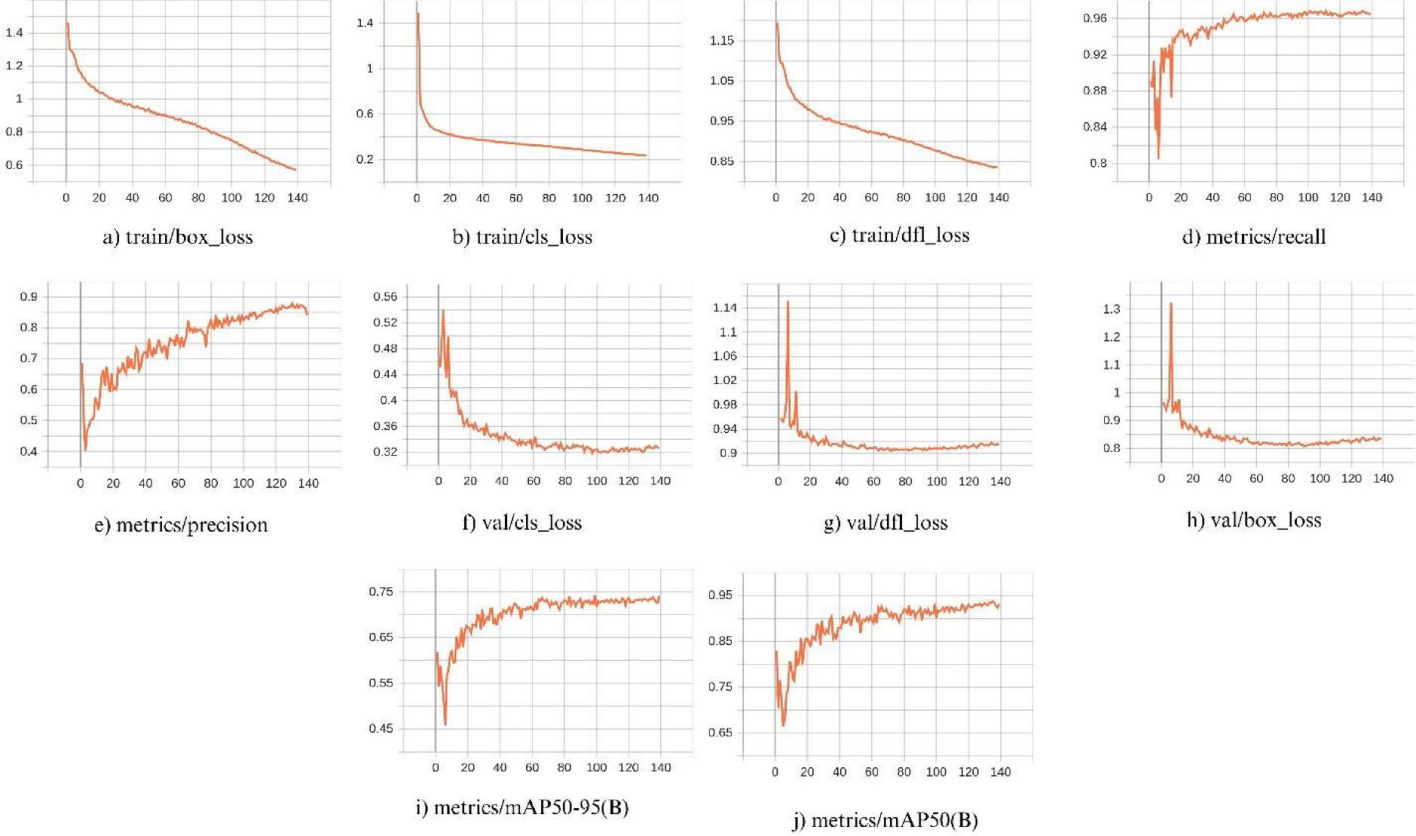
Performance of fine-tuned object detection model (YOLOv8x) for insect detection. Plots (**a**–**c**) on the top row present box loss, classification loss, and distribution focal loss; plots (**d**) and (**e**) on the top row present precision and recall. Plots (**f**–**h**) on the bottom row show validation box loss, validation classification loss, and validation distribution focal loss plot respectively. Plots (**i**) and (**j**) on the bottom row present mean average precision (mAP) over 140 epochs under different Intersections over Unions (IoUs).

Figure 15 presents the performance of the object detection model (Yolo-pest) in locating the coordinates of the object and how well the predicted bounding box covers the entire object. The model exhibited a high inference time of 8157.6 ms per image, rendering it an unreliable solution for the smart trap. Therefore, the decision was made to export the model in ONNX (Open Neural Network Exchange) format after applying the simplify optimization technique.

**Fig. 16:**
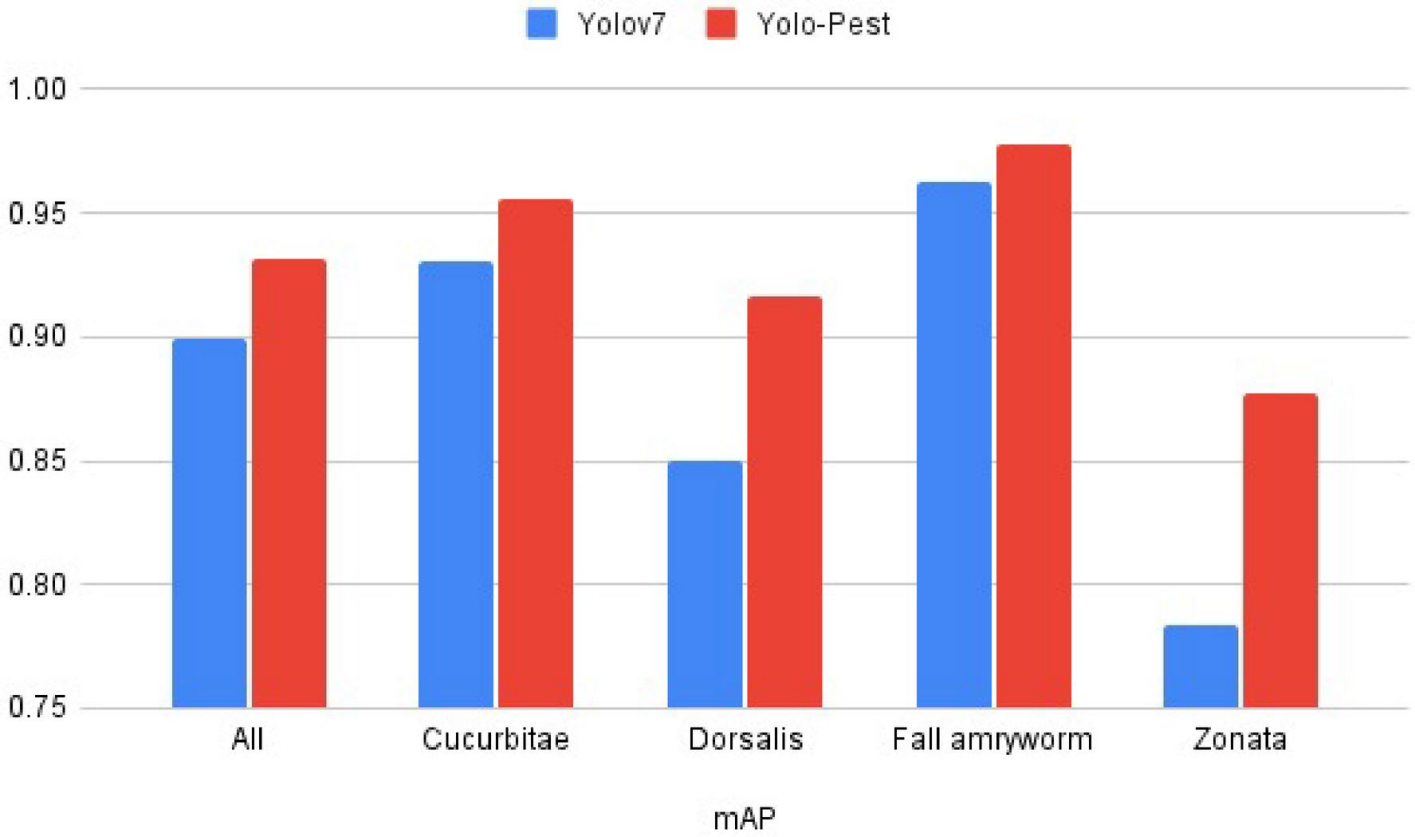
Comparision of yolov7 and yolo-pest on the validation dataset.

Onnx is an open and interoperable machine learning model format designed to facilitate the compatibility of different frameworks, the key features of Onnx format are its interoperability, optimization, and versioning. The optimization technique removes unnecessary layers of the trained model without compromising its accuracy^48^. A comparison was conducted between YOLOv7 and the proposed YOLO-pest models for the detection of selected pest species. The result of experiments suggests that Yolo-pest exhibited the fastest training time of 13.67 h, followed by YOLOv7 at 30 h. This suggests that Yolo-pest is more efficient in terms of computational resources and time. In terms of performance on object detection, Yolo-pest achieved the highest mAP of 0.974 on the testing dataset, indicating better performance across multiple classes (see Table 3 for numeric and Fig. 16 for graphical illustration). The model also showed better robustness by effectively balancing the recall and precision.

**Fig. 17:**
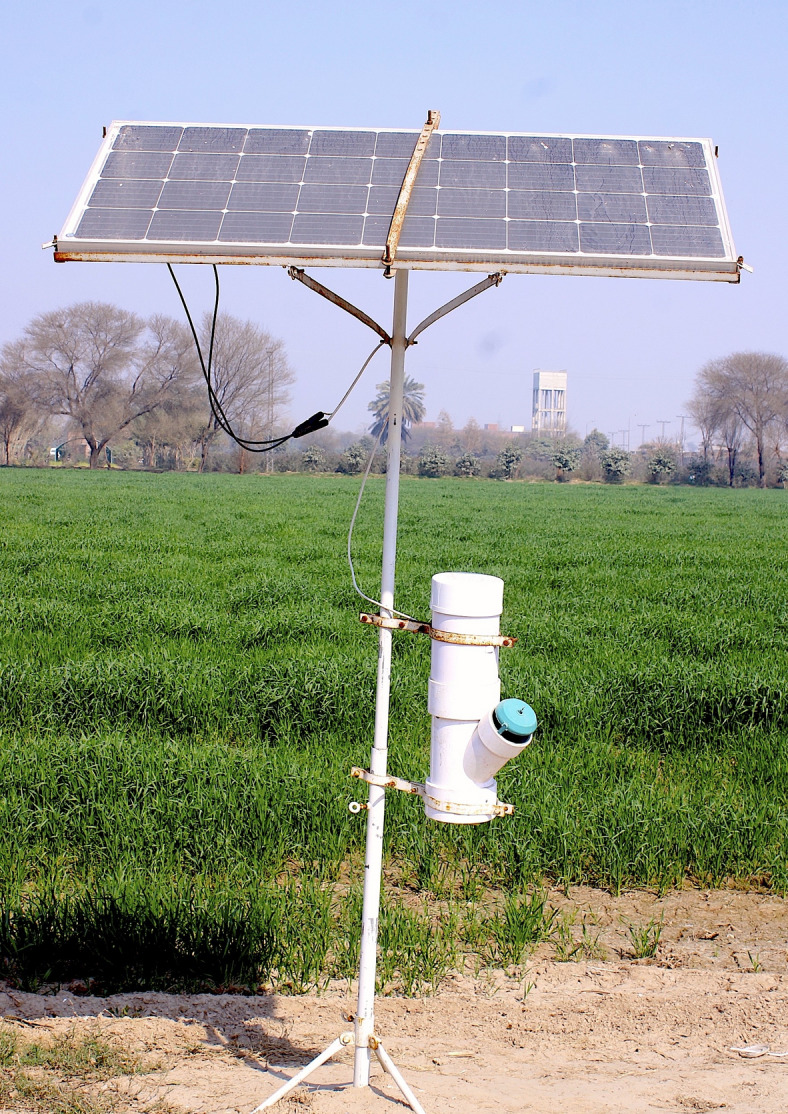
Smart trap deployed in mango orchard under arid climatic conditions.

The operational efficacy of the smart trap and the trained model was evaluated by replicating the deployment of the smart traps (Fig. 17) at multiple locations in mango orchards in the summer season for four months starting from May 2022 to September 2022, and in maize field to evaluate the model on fall-armyworms for three months from March 2023 to May 2023. The trap was securely clamped to a steel pole, with the solar panel oriented toward the northwest to optimize exposure to sunlight. The solar panel charges the rechargeable lithium battery that was used to power up the smart trap. With these configurations, the smart traps can operate continuously for 3 days without charging and indefinitely with charging. Farmers make decisions about control measures based on a daily count of fruit flies and fall armyworms that is made available on the customized mobile application.

## Results

**Fig. 18:**
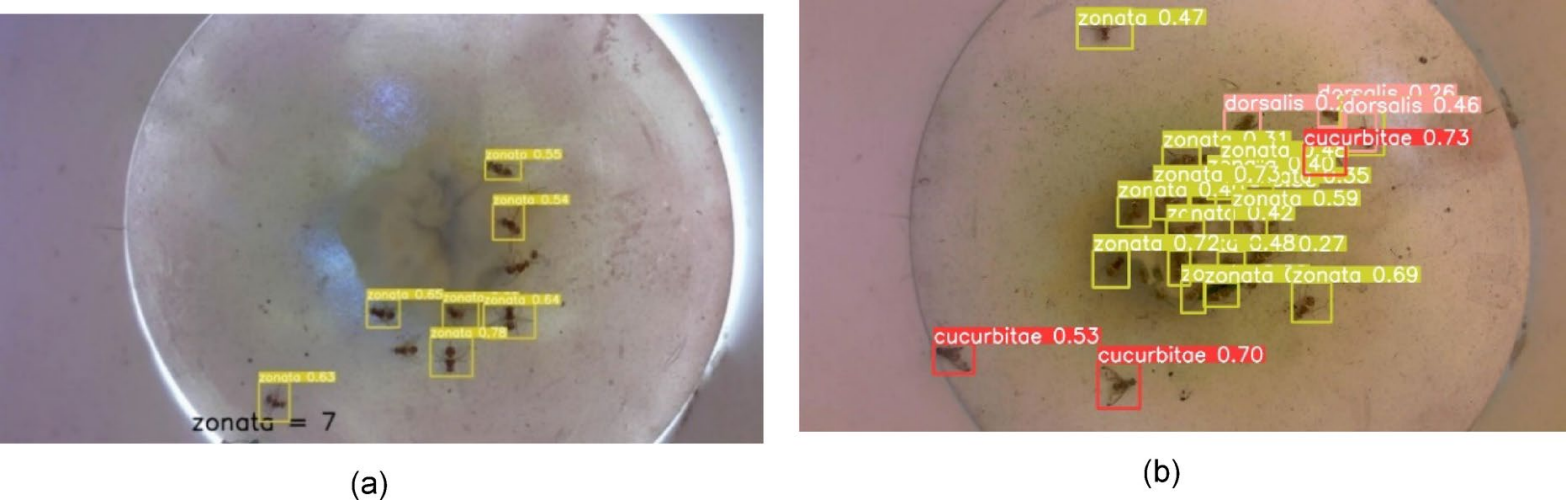
(**a**): Detection of fruit fly *B. zonata* in real-time images captured by smart trap in sunlight installed in open field environment. (**b**) Detection of fruit fly zonata on real-time images captured by smart trap in the evening installed in open field enviornment.

The model was evaluated using real-time images captured by the smart trap, as well as images obtained from high-quality cameras in laboratory environments. The testing images were never seen by yolo-pest object detection model before. The results of the real time fruitfly detection showed that model performed reasonablly well for the images collected either during sunshine hours and even later of the day during evening hours as the smart trap was installed in open field environment. The confidence level of the model ranges between 54 and 78% for the detection of fruit fly (B. zonata) in real-time images captured by smart trap during sunshine hours (Fig. 18a). While, the confidence level of the model found also good for the images collected in evening hours where it ranges between 27% (occluded insect) to 73% for detection of fruit fly zonata (Fig. 18b).

**Fig. 19:**
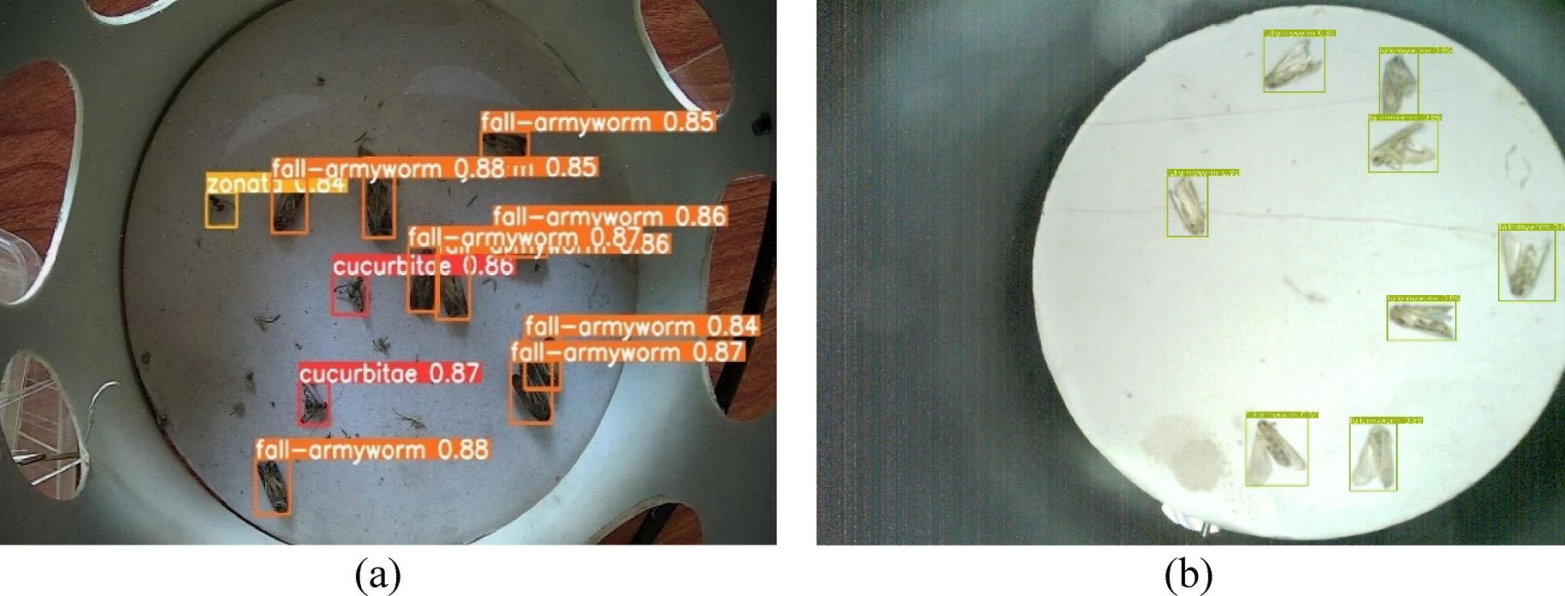
(**a**) Detection of multiple fall-armyworms and fruit flies using the object detection model in the laboratory setting under natural day light. (**b**) Detection of multiple fall-armyworms in the laboratory setting under flash-assisted light.

Similirarly, the results also showed the good model performance for detection of multiple fall-armyworms and fruit flies in the laboratory conditions under natural day light, where the model confidence level ranges between 84 and 88% foir tested images (Fig. 19a). While the confidence level of the model also found reasonably good as it ranges between 86 and 88% for detection of multiple fall-armyworms in the laboratory setting under flash-assisted light (Fig. 19b). The variation in lighting sources helps evaluate the model’s performance under different conditions, ensuring robustness in both natural and controlledconditions. Each pest is detected by a bounding box along with a confidence level that indicates the probability, with which the estimation of the location of the detected species is true for the entire dataset. In laboratory environments, the smart traps exhibit confidence levels between 84% and 88%. This high range indicates strong reliability under controlled conditions where variables are minimized and conditions are ideal for the sensors and detection algorithms to performs the functions optimally. In contrast, the confidence levels in field settings range from 27 to 78%. This lower and wider range reflects the challenges and variability posed by real-world conditions. Factors such as environmental noise, fluctuating weather conditions, and varying lighting conditions significantly affect the performance of the traps. This variability in confidence levels highlights the potential for decreased detection accuracy in field settings, impacting the overall effectiveness of the system.

Table 5 illustrates various operational conditions and performance metrics of smart traps in laboratory settings (indoor environment) and open field environments. The table covers factors such as power supply that is stable in the lab but relies on variable solar power in the field, and internet availability that is consistent in the lab but potentially limited in the field. Temperature and humidity are controlled and consistent in the lab, whereas they vary to extreme ranges in the field affecting trap performance and maintenance needs. A comparison was conducted between battery life under constant charging conditions and performance in less reliable field environments, as well as an analysis of pest capture rates. Capture rates were observed to be higher in controlled environments, while a decline occurred in natural settings due to competing attractions, such as food sources for insects. The table also highlights trap durability concerns, that experience minimal wear and tear in lab settings as compared to uncontrolled harsh field conditions. Data accuracy and calibration are easily managed and maintained in the laboratory as compared to the field environment. The smart traps automate pheromone traps designed to attract specific insects. In the open field installations, No non-target species were observed in the traps, with the exception of a few ants and spiders.These were effectively ignored by the yolo-pest model, demonstrating the model’s robustness and specificity in distinguishing between target and non-target species.

**Table 5:** Comparison of smart trap performance attributes in laboratory and open field environments.

Factor	Laboratory setting	Open field environment	Smart traps performance in lab	Smart traps performance in the field
Power Supply	Stable, continuous access to electricity.	Rely on batteries and solar panels.	100% operational uptime.	85% operational uptime due to variable sun exposure.
Internet	High-speed, reliable connectivity.	Variable; may be limited or weak.	Data transmission every 5 min.	Data transmission every 120 min or as signal permits.
Temperature	Controlled, 23 °C (73 °F).	Extreme temperature ranges from 1 °C (-17 °F) to 50 °C (10 °F).	Consistent capture rate.	10% decrease in capture efficiency in extreme temperatures.
Humidity	Maintained at 40–50%.	Ranges from 30–90%.	No impact on functionality.	15% reduction in sensor accuracy at high humidity levels.
Maintenance	Regularly scheduled, easily accessible.	Irregular, dependent on weather and location.	5% downtime for maintenance.	20% downtime for maintenance issues.
Battery Life	Constantly charged, no significant drain.	Variable, reliant on solar charging.	Always fully charged.	3 days of continuous operation without sunlight.
Pest Capture Rate	High, consistent due to controlled environment.	Variable, dependent on environmental factors.	95% capture efficiency.	75% capture efficiency.
Durability	Not exposed to harsh elements.	Exposed to rain, wind, and other elements.	Minimal wear and tear.	Increased wear and tear, reduced lifespan by 30%.
Data Accuracy	High, controlled conditions.	Potential interference from environmental factors.	97% accurate data.	92% accurate data.
Calibration	Easy to calibrate regularly.	Difficult to perform regular calibration.	100% accurate calibration.	80% accurate calibration over time.
Non-target Species	Minimal interaction due to controlled conditions.	Minimal interaction because pheromones attract target species only.	5% false positive rate.	5% false positive rate.

**Fig. 20:**
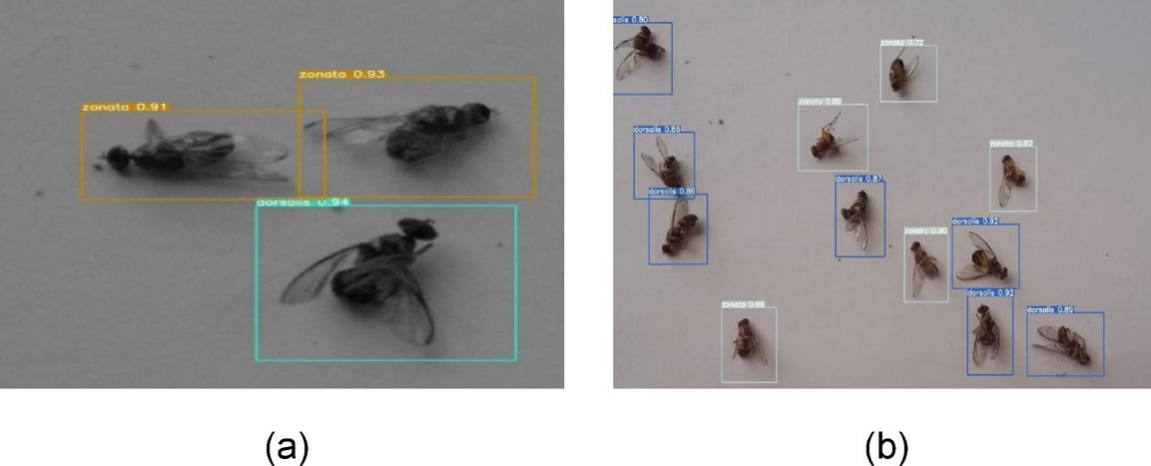
(**a**) Validation of object detection model on grayscale image. (**b**) Validation of object detection model on colored image.

Results were further validated by testing on both grayscale and colored images of fruit flies. The model performed equally well on a completely new set of images. Figure 20a shows the validation of trained model on a grayscale image, and Fig. 20b shows the validation of a colored image that contains multiple fruit flies.

Both raw and processed images of pests captured by the smart trap camera are stored on the server and become part of the dataset for life-long learning. The dataset can be used by researchers and entomologists to conduct various studies related to fruitfly species and fall armyworms. The dataset also includes values of temperature and humidity with date and time that can be used to study the effect of environmental factors on the population density of flying insect pests.

**Fig. 21:**
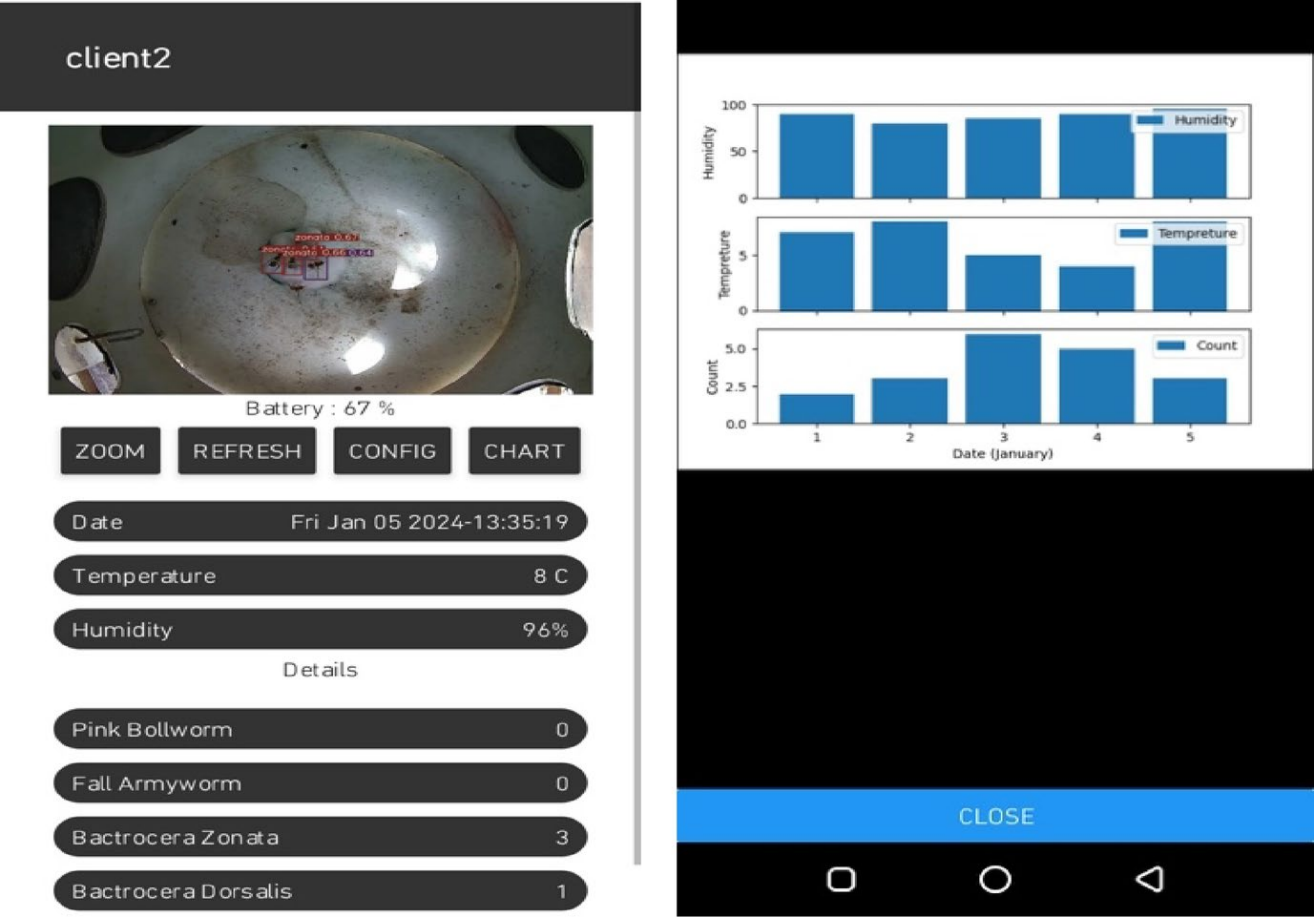
Graphical user interface of mobile application.

A specially designed mobile application has been developed to visualize the results of species detection and identification in real-time without visiting the farm (Fig. 21). The application shows real-time images from the trap, date/time, precise temperature, and humidity in the field. It also shows the insect count and charts showing the daily weather pattern and trend of population density. Using this application, the farmer can visualize the status of insect count, and species of insect pests along with real-time data of local temperature and humidity whenever and wherever required. This mobile app is flexible and easy to use by farmers. Only the registered user can log into the system. After logging in, the user can access the data streaming from all traps installed in the registered orchard. The mobile application shows real-time images from the trap, date/time, precise temperature, and humidity in the field. It also shows the insect count and charts showing the daily weather pattern and trend of population density.

## Discussion

Traditional practices for monitoring of insect pests in crop, vegetable and orchards are time-consuming, ineffective, and costly. Farmers face significant crop damage and quality loss annually due to these pests which is also a major hurdle in the exports of grains, seeds, fruits and food products. There is an urgent need for digital interventions and precision technologies for resoruce monitoring and optimization in agriculture for environmental protection and sustainability under changing climatic scenarios. Further, there is a need to intervene in technology to continuously and real time monitor the damages of flying insect pests at the farm with minor nohuman involvement. This studypresents a digital technology like smart trap for continuous monitoring of three major species of fruit flies (*Bactrocera zonata*,* Bactrocera dorsalis*,* and Bactrocera cucurbitae*) and fall-armyworm in maize crop at farms scale for timely update the farmers about insect pest hotspots (locations) via alerts through mobile applciation for timely and effective decision making to control the insect pests.

The traditional ways of insect monitoring are by using sticky tapes or pheromone traps in the field that need to be visited regularly. These techniques are mostly ineffective. There have been a few attempts to automate these traps by attaching a camera to take images regularly, however, most of the studies are too specific to be used in different environments and insects. Many automated traps do not consider the extreme environmental conditions in open fields and other limitations such as power backup and network connectivity. Although, the past related studies like Chen et al.^34^ also developed the Yolov4 algorithm for detecting pests (*Mealybugs*, *Coccidae*, and *Diaspididae)* and claimed to achieve 100% accuracy on the test dataset. But, the dataset was only limited to 600 images while the model was performed and test for 35 iamges, while the current studies focused on small insect pest in different conditions by utilizing a big dataset of the images. Likewie in an other previous study, Wen et al.^35^ proposed the Pest-YOLO model which was trained and evaluated on the Pest24 dataset, which includes more than 20,000 images with over 190,000 instances labeled and categorized into 24 classes. The results show that Pest-YOLO achieved 69.59% for mAP and 77.71% for mRecall on Pest24, while the current study secured better accuracy and reliability using the different big data set in contrasting environments. Similarly, Yang et al.^36^ proposed Maize-yolo which is designed to detect maize pests. The model was trained and evaluated on the IP13 dataset which contains only 4533 images. Maize-yolo achieved an accuracy of 0.763 mAP. In comparison, findings from the current study revealed that smart trap achieved 97% average detection accuracy from testing dataset ‘FourPest’, containing 36,822 instances (cucurbitae: 6619; dorsalis: 11472; zonata: 10095; fall-armyworm: 8636) in an open agricultural field conditions .The smart trap is an automation of a pheromone trap that enables real-time monitoring of the fruit flies’ and fall armyworm population in the fields. The trap is designed in consultation with entomologists, farmers, and field experts based on insect behavior, crop season, weather parameters, and open field environmental conditions. With the help of multi-band antennas, it can transmit real-time data from cellular networks to the server for object detection. The trap is weather-resilient and can run on solar power with a minimum cost that is affordable to average farmers.

While the smart trap system has demonstrated effectiveness, while certain limitations need further consideration. The effectiveness of the system may vary concerning factors such as insect size, behavior, and environmental conditions. Additionally, the reliance on pheromones and the specific attractants used may limit its suitability for a broader spectrum of pests. This study primarily focused on flying insect pests, and the system’s performance with other insect life stages warrants further investigation. Although IoT sensors and trap housing are prone to harsh environmental conditions, extreme temperatures or heavy rainfall may impact the system’s performance and durability. The reliance on battery power poses challenges, particularly in areas with limited sunlight for solar charging, potentially affecting the system’s continuous operation. Furthermore, consistent internet connectivity is crucial for real-time data transmission and remote monitoring; however, in remote or poorly connected areas, this dependency may pose a limitation.

Addressing these challenges requires technological advancements in robust hardware design, power optimization, and alternative communication strategies, ensuring the system’s resilience in diverse and challenging field conditions. Ongoing research and development are essential to enhance the system’s adaptability and reliability in varying environments. Further, we are working towards maximizing the efficiency of the trap by looking for alternative sources of the network. Current study demonstrated the functionality of a smart trap for fruit fly and fall armyworm monitoring in maize crop, while another study is also in progress to further evaluate and train the model and smart trap for advanced consideration like to monitor other major flying insect pests such as Pink Bollworm moths that badly affect cotton crops and lint qulaity.

The smart trap demonstrates improved efficiency in making informed decisions to control pests’ growth to avoid crop damage. The object detection model classified the insects, based on the difference in their appearance and individually counted the insects present in the trap. If the population count reaches the Economic Threshold Level (ETL)^49^ set by the government, the farmer receives an alert notification to take precautionary measures to avoid crop damage. The proposed object detection model achieves an average accuracy of 94% on an image dataset of three fruit fly species and FAW collected from two sources: insect insect-rearing laboratory and a trap installed in the maize field. The results suggest that AI, IoT, and mobile applications are a feasible solutions for monitoring pests in real time. This study will be further expanded to analyze the relationship between the population density of fruit flies with environmental factors and climate change. The advantages of proposed insect monitoring smart traps extend beyond pest control; they prove invaluable for early detection and surveying, especially concerning invasive species. The potential to interconnect traps across various sites opens avenues for creating networks at local, regional, country, continental, and global scales^50^. These interconnected networks, when integrated into a ‘Big Data’ system, can incorporate diverse environmental and geographical parameters, including geo-coordinates, weather trends, forecasting models, and control techniques. Such integrated systems empower growers, agriculturalists, and forest managers to optimize pest control measures through technology-driven approaches in conventional and organic management programs. In the future, the device will also be connected to a spraying system for automatic precise pesticide application when the fruit fly count reaches a maximum threshold. This will enable effective and environmentally friendly pest control by minimizing the need for widespread pesticide spraying.

## Conclusion

This research presents a comprehensive approach to monitoring and managing flying insect pests in mango orchard and maize crop settings through the development of a smart trap and Yolo-pest that is a fine-tuned the YOLOv8x object detection model. The results of the study are validated through extensive testing, demonstrate the exceptional performance of the fine-tuned YOLOv8x model in detecting small insects, across diverse environmental conditions. The smart trap, deployed in a mango orchard and maize field under arid climatic conditions, showcased robust capabilities in capturing real-time images and detecting multiple fruit flies and fall-armyworms. The validation process on grayscale and coloured images further confirmed the model’s consistent accuracy in identifying insect pests. The establishment of a dataset enriched with temperature, humidity, date, and time values adds substantial value to ongoing research in entomology, offering a resource for studying the correlation between fruit fly species and environmental factors.

The specially designed mobile application enhances the practical utility of theresearch, providing a user-friendly interface for real-time visualization of species detection and identification. Farmers can remotely access crucial data, including insect count, pest species identification, and environmental conditions, empowering them with valuable insights for proactive pest management. The application’s flexibility and accessibility, restricted to registered users, ensure a secure and efficient tool for informed decision-making in agriculture.

The successful development and evaluation of the smart trap and the fine-tuned YOLOv8x model not only contribute to the field of agricultural pest management but also hold promising economic, environmental, and health-related implications. By offering precise and efficient monitoring tools, this research is positioned to revolutionize pest control strategies, minimizing the use of pesticides, reducing costs, and promoting sustainable farming practices. Wayforward of the study is furtherintegration of such technologies marks a significant step towards achieving a harmonious balance between agricultural productivity and environmental conservation.

## Data Availability

A sample of the dataset is made publicly available at: https://data.mendeley.com/datasets/hgz2n5jxhp/1. The complete dataset will be shared by the corresponsing authors on request.
